# Standardization of neutrophil CD64 and monocyte HLA-DR measurement and its application in immune monitoring in kidney transplantation

**DOI:** 10.3389/fimmu.2022.1063957

**Published:** 2022-11-23

**Authors:** Bo Peng, Min Yang, Quan Zhuang, Junhui Li, Pengpeng Zhang, Hong Liu, Ke Cheng, Yingzi Ming

**Affiliations:** ^1^ Transplantation Center, The Third Xiangya Hospital, Central South University, Changsha, China; ^2^ Engineering and Technology Research Center for Transplantation Medicine of National Health Commission, Changsha, China

**Keywords:** nCD64, mHLA-DR expression, infection, sepsis, kidney transplantation, immune monitoring, prognosis

## Abstract

**Background:**

Infections cause high mortality in kidney transplant recipients (KTRs). The expressions of neutrophil CD64 (nCD64) and monocyte HLA-DR (mHLA-DR) provide direct evidence of immune status and can be used to evaluate the severity of infection. However, the intensities of nCD64 and mHLA-DR detected by flow cytometry (FCM) are commonly measured by mean fluorescence intensities (MFIs), which are relative values, thus limiting their application. We aimed to standardize nCD64 and mHLA-DR expression using molecules of equivalent soluble fluorochrome (MESF) and to explore their role in immune monitoring for KTRs with infection.

**Methods:**

The study included 50 KTRs diagnosed with infection, 65 immunologically stable KTRs and 26 healthy controls. The blood samples were collected and measured simultaneously by four FCM protocols at different flow cytometers. The MFIs of nCD64 and mHLA-DR were converted into MESF by Phycoerythrin (PE) Fluorescence Quantitation Kit. The intraclass correlation coefficients (ICCs) and the Bland-Altman plots were used to evaluate the reliability between the four FCM protocols. MESFs of nCD64 and mHLA-DR, nCD64 index and sepsis index (SI) with the TBNK panel were used to evaluate the immune status. Comparisons among multiple groups were performed with ANOVA one-way analysis. Receiver operating characteristics (ROC) curve analysis was performed to diagnose infection or sepsis. Univariate and multivariate logistic analysis examined associations of the immune status with infection.

**Results:**

MESFs of nCD64 and mHLA-DR measured by four protocols had excellent reliability (ICCs 0.993 and 0.957, respectively). The nCD64, CD64 index and SI in infection group were significantly higher than those of stable KTRs group. Patients with sepsis had lower mHLA-DR but higher SI than non-sepsis patients. ROC analysis indicated that nCD64 had the highest area under the curve (AUC) for infection, and that mHLA-DR had the highest AUC for sepsis. Logistic analysis indicated that nCD64 > 3089 and B cells counts were independent risk factors for infection.

**Conclusion:**

The standardization of nCD64 and mHLA-DR made it available for widespread application. MESFs of nCD64 and mHLA-DR had good diagnostic performance on infection and sepsis, respectively, which could be promising indicators for immune status of KTRs and contributed to individualized treatment.

## Introduction

Kidney transplantation (KTx) is currently regarded as the most effective therapeutic approach for end-stage renal disease (ESRD) (1). Although the graft and patient survivals post KTx have been enhanced greatly in recent decades, infection is still the second leading cause of mortality in kidney transplant recipients (KTRs) (roughly 15% – 20%) (1). More seriously, KTRs with sepsis, which is characterized by dysregulation of the immune response following infection, have even higher mortality rate (2–4). Due to the intense induction therapy and long-term maintenance immunosuppressive regimen, the pathophysiology of KTRs with infection is heterogeneous and comprises both hyperinflammatory and immunosuppressive phenotypes. Although some biomarkers have been reported to predict infection in KTRs, it is still pivotal to identify optimal immunologic parameters to assess host immune status for early diagnosis and individualized treatment (5, 6).

A wide range of studies have revealed that elevated expression of neutrophil CD64 (nCD64) is linked to pro-inflammatory reaction, while decreased expression of monocyte HLA-DR (mHLA-DR) is linked to immunosuppression (7). CD64, a high affinity immunoglobulin (Ig)-G Fc receptor (FcγR), is characterized by a rapid and intense increase in expression on neutrophils in response to infection or pro-inflammatory cytokines (8). Some literatures have revealed that nCD64 is an effective biomarker for the diagnosis of infections, the assessment of sepsis severity and the prediction of prognosis (9, 10). Meanwhile, mHLA-DR, which presents antigens to T cells to initiate the adaptive immune response, is also an important indicator to assess the immune status (10). Several studies have found that low expression of mHLA-DR is associated with increased risk of acquiring secondary infections and mortality (10, 11). The predictive value of mHLA-DR in prognosis on various conditions, including sepsis (10, 11), nosocomial infection (12), SARS-CoV-2 infection (13) and solid organ transplantation (14, 15), has also been verified by various clinical investigations. Our previous study indicated that KTRs with pneumonia appeared lower expression of mHLA-DR and higher expression of nCD64, which were important parameters to predict the prognosis of pneumonia using machine learning models (16).

Although the value and effect of nCD64 and mHLA-DR for immune monitoring have been validated, it is difficult for horizontal comparison. In the majority of previous studies, the expression of nCD64 and mHLA-DR were assessed by the mean fluorescence intensity (MFI) *via* flow cytometry (FCM), which was a relative value. MFI is determined not only by the expression intensity but also by the flow cytometer settings and the antibody selected, thus limiting its widespread application. It is urgently required to establish standardized quantification of nCD64 and mHLA-DR for further clinical application.

In this study, we standardized the measurement of nCD64 and mHLA-DR by converting their MFI values into molecules of equivalent soluble fluorochrome (MESF) (17). Furthermore, a prospective longitudinal analysis of the standardized nCD64 and mHLA-DR in KTRs was performed to explore their performance in immune monitoring.

## Materials and methods

### Study design and population

**Figure 1:**
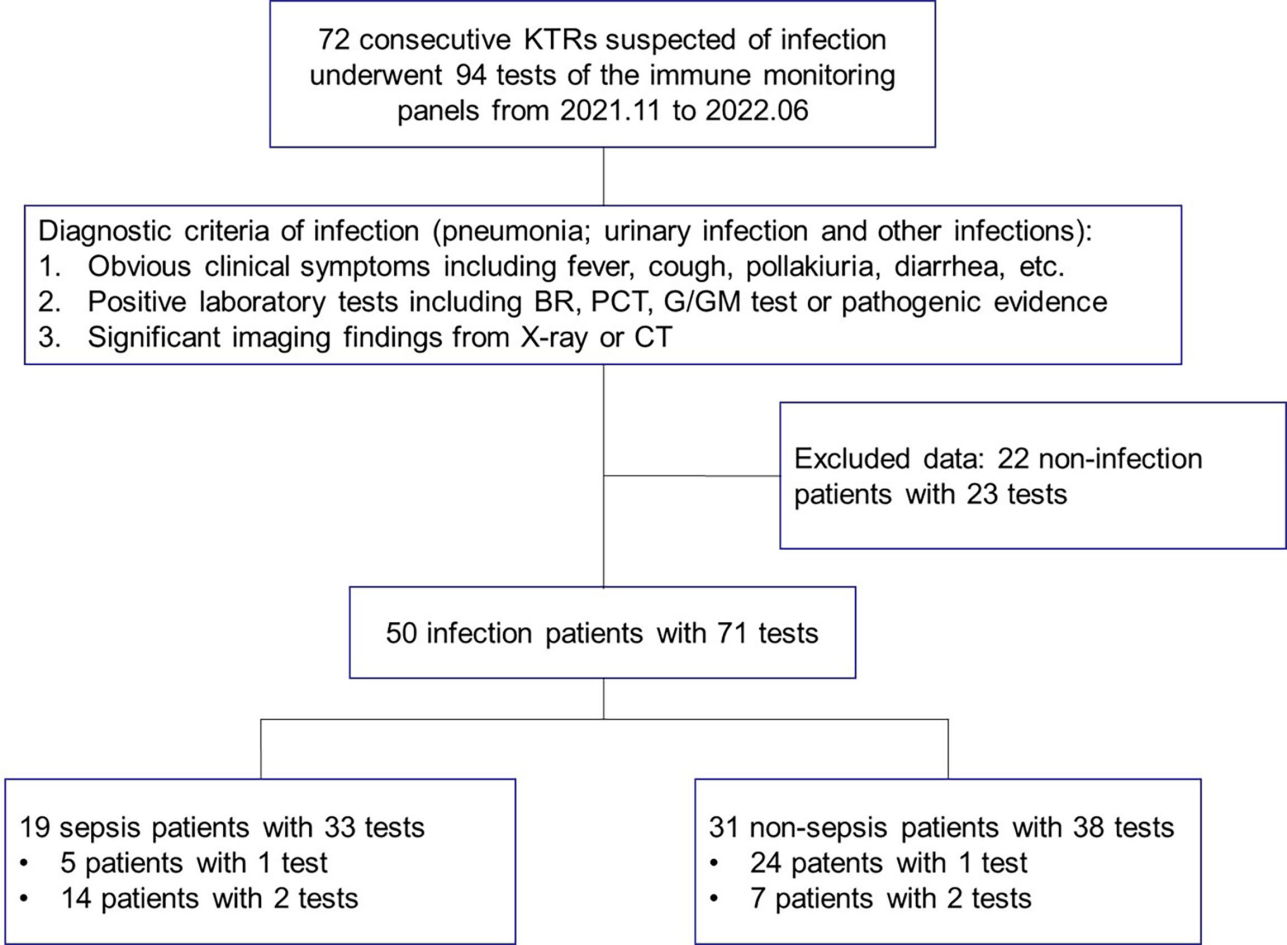
The study flow and diagnostic criteria of infection. 72 KTRs with 94 tests of immune function panels were first enrolled. 50 KTRs conformed of infection were further classified into the sepsis group and non-sepsis group according to the definition for sepsis. KTRs, kidney transplant recipients; BR, blood routine examination; PCT, serum procalcitonin; G/GM test, (1-3)-beta-D-glucan or galactomannan test; CT, computed tomography.

In this prospective, longitudinal, observational study, 72 consecutive KTRs suspected of infection were recruited from the Transplantation Center, The Third Xiangya Hospital, Central South University from November 1, 2021, to June 31, 2022. The immune monitoring panels including both the standardized nCD64 and mHLA-DR panels and the TBNK panel were performed at two time points, namely, 1 – 3 days and 5 – 8 days post admission. All the patients were 18 – 65 years old, and those who did not meet the diagnostic criteria of infection were excluded (**Figure 1**). The stable outpatient KTRs (n = 65) and another group of healthy controls (HCs, n = 26) were also recruited and received the immune monitoring panels once. The inclusion criteria for stable KTRs were as followings: (1) more than 3 months post KTx; (2) no signs of rejection, tumor or infection; (3) stable allograft function (the creatinine less than 171 μmol/L). Informed consent was obtained for each patient, and the study was approved by the Institutional Review Board of Third Xiangya Hospital, Central South University (No. 21176).

All KTRs received KTx from donation after citizens’ death (DCD) after 2012 or from close family members. The allografts from DCD were attributed by the China Organ Transplant Response System. All transplants performed were approved by the Ethics Committee of the Third Xiangya Hospital, Central South University. Routine induction therapy included anti-thymocyte globulin (ATG, 1.00 mg/kg daily for 3 days) or basiliximab (20 mg at days 0 and 4), and the standard triple immunosuppressive regimen, namely, calcineurin inhibitor (CNI), mycophenolate mofetil/entericcoated mycophenolate sodium, and corticosteroid was given as a maintenance regimen.

### Diagnostic criteria of infection and sepsis

Diagnostic criteria of infection (pneumonia, urinary infection or other infections) were as followings: (1) obvious clinical symptoms including fever, cough, pollakiuria, diarrhea, etc.; 2, positive laboratory tests including blood routine examination, serum procalcitonin, (1-3)-beta-D-glucan/galactomannan test (G/GM test) or pathogenic evidence; 3, significant imaging findings from X-ray or computed tomography (CT) (**Figure 1**). Sepsis was defined according to The Third International Consensus Definitions for Sepsis and Septic Shock (Sepsis-3), and organ dysfunction was defined as Sequential Organ Failure Assessment (SOFA) score of 2 points or more (4). Due to the fact that stable KTRs might maintain a relatively poorer renal function than the HCs, strict assessment of renal function according to the SOFA score would overestimate the severity of infection. Therefore, only KTRs with the creatinine equal to or greater than 171 μmol/L (SOFA score ≥ 2 points for renal function), or KTRs with obvious increase of creatinine during infection, were regarded as renal dysfunction for SOFA scoring.

### Standardization protocol of nCD64 and mHLA-DR

Two panels were used to detect the MFIs of nCD64 and mHLA-DR using the following fluorochrome-conjugated monoclonal antibodies: anti-CD45-KRO (clone 22202012, Beckman Coulter), anti-CD14-APC (clone 22205028, Beckman Coulter), anti-HLA-DR-PE (clone Immu-357, Beckman Coulter) and anti-CD64-PE (clone 200053, Beckman Coulter). The nCD64 panel contained the anti-CD45 and anti-CD64 antibodies, while the mHLA-DR panel contained the anti-CD14 and anti-HLA-DR antibodies. Briefly, 50 μl whole blood from the identical EDTA anticoagulation tube was used for detection in each panel. After erythrolysis, cells and monoclonal antibodies were incubated in the dark for 15 minutes. After washing and resuspending, samples were detected and the MFIs of nCD64 and mHLA-DR were acquired with the two panels, respectively. The gating strategy was shown in the **Supplementary Figure 1**.

A total of four settings on two flow cytometers (BD FACSCanto II and Beckman Coulter DxFlex) were used to evaluate the reliability of the standardization protocol of nCD64 and mHLA-DR. On BD FACSCanto II, the voltage of PE channel (detecting the MFIs of nCD64 and mHLA-DR) was set at high, medium and low levels. On Beckman Coulter DxFlex, a fixed, proper voltage of PE channel was set. The MFIs of nCD64 and mHLA-DR for the identical sample were acquired under these four settings.

Then, the MFIs were converted to MESFs using PE Fluorescence Quantitation Kit (BD Quantibrite™ Beads, BD Biosciences). In this kit, beads were conjugated with calibrated four levels of PE molecules. The kit was run under the above four settings. A linear regression of Log_10_ MFI against Log_10_ PE molecules per bead (namely the MESF value) was performed. With the parameters of the linear regression, the MFIs of nCD64 and mHLA-DR were converted to MESFs. The mean value of the four results under the four settings mentioned above was used for further analysis.

### nCD64 index and sepsis index

The formula for calculating the nCD64 index was shown as following: nCD64 index = (nCD64/lymCD64)/(mCD64/nCD64) (9). lymCD64 was the expression of CD64 on lymphocytes and mCD64 was expression of CD64 on monocytes. Sepsis Index (SI) was shown as following: SI = nCD64/mHLA-DR×100 (18). All parameters were calculated with the MESF values.

### TBNK panel and lymphocyte counts

Another panel for immune monitoring was BD Multitest 6-color TBNK reagent with BD Trucount tubes, which identified the percentages and absolute counts of CD3^+^CD4^+^ T cells, CD3^+^CD8^+^ T cells, CD19^+^ B cells and NK cells. This panel was performed according to the manufacture’s instruction and analyzed by BD FACSCanto clinical software (BD Biosciences, San Jose, CA, USA).

### Statistical analysis

Continuous data were presented as the mean ± standard deviation (SD) or median with interquartile range (IQR), and were compared using Student’s t test or Welch’s t test, where appropriate. Categorical data were compared using Pearson’s chi-squared (χ^2^) test or Fisher’s exact test, where appropriate. Comparisons among multiple groups were performed with ANOVA one-way analysis. The least significant difference (LSD) test was used for back testing of multivariate ANOVA. The intraclass correlation coefficients (ICCs) and the Bland-Altman plots were used to evaluate the reliability of standardization protocol of nCD64 and mHLA-DR. Receiver operating characteristics (ROC) curve analysis was performed to evaluate the diagnostic efficiency of infection or sepsis. Univariate and multivariate logistic analysis examined the associations of immune status and infection. Statistical analysis was performed using SPSS version 22.0 (SPSS, Inc., Chicago, IL, USA) and GraphPad Prism 9.0. P < 0.05 was considered to be statistically significant.

## Results

### Patient characteristics

Seventy-two KTRs suspected of infection, 65 stable KTRs and 26 HCs were recruited and 185 tests of immune monitoring panels were performed. The immune monitoring panels included both the standardized nCD64 and mHLA-DR panels and the TBNK panel. Based on the diagnostic criteria of infection, 50 patients were conformed diagnosis of infection. Within the infection group, 19 patients were further categorized as the sepsis subgroup according to the definition of sepsis. Fourteen of the 19 sepsis patients received the immune monitoring panels twice, but 5 patients received only once due to their limited hospital stay. The study flow and details were shown in **Figure 1**.

**Table 1** shows the clinical characteristics of the HCs and KTRs. There was a significant difference of age between the HCs and KTRs (P < 0.001), but no difference between the stable and infection groups (P = 0.112). More male patients were in the stable group (P = 0.036). Compared with the stable group, the infection group had lower lymphocyte count and higher serum creatinine at admission (P < 0.001 and P < 0.001, respectively). There was no statistical difference of donor source, calcineurin inhibitor, time from transplant to 1st test, neutrophil count or white blood cell (WBC) counts between the stable and infection groups.

**Table 1:** Clinical characteristics of the HCs and KTRs.

Parameters	HCs (n = 26)	KTRs				*P* value^#^
		All KTRs (n = 115)	Stable KTRs (n = 65)	Infection KTRs (n = 50)	*P* value^*^	
Male recipient, n (%)	15 (57.7%)	70 (60.9%)	45 (69.2%)	25 (50.0%)	0.036	0.428
Age, yrs ± SD	31.31 ± 10.65	42.02 ± 12.16	44.55 ± 9.56	44.30 ± 13.05	0.112	< 0.001
Donor, n (%)	NA				0.743	NA
DCD		83 (72.2%)	49 (69.2%)	34 (68.0%)		
Relative		32 (27.8%)	16 (30.8%)	16 (32.0%)		
Infection site, n (%)	NA	NA	NA		NA	NA
Pneumonia				27 (54%)		
Urinary infection				17 (34%)		
Other infection				6 (12%)		
Calcineurin inhibitor, n (%)	NA				0.412	NA
Tacrolimus		108 (93.9%)	60 (92.3%)	48 (96.0%)		
Cyclosporine A		7 (6.1%)	5 (7.7%)	2 (4.0%)		
Time from transplant to 1st test, months ± SD	NA	62.64± 52.90	69.86 ± 48.69	53.26 ± 57.06	0.295	NA
WBC (10^9^/L)	NA	7.29 ± 3.27	6.78 ± 2.10	7.94 ± 4.28	0.076	NA
Neu (10^9^/L)	NA	7.02 ± 10.83	6.48 ± 11.18	7.74 ± 10.43	0.537	NA
Lym (10^9^/L)	NA	1.32 ± 0.74	1.64 ± 0.69	0.90 ± 0.58	< 0.001	NA
Cr admission (μmol/L)	NA	143.73 ± 88.42	121.91 ± 54.94	172.10 ± 113.10	< 0.001	NA

*Comparison between the stable KTRs and the infection KTRs.

^#^Comparison among the three groups.

HCs, healthy controls; KTRs, kidney transplant recipients; SD, standard deviation; DCD, donation after citizens’ death; WBC, white blood cell counts; Neu, neutrophils count; Lym, lymphocyte counts; Cr, serum creatinine; NA, not available.

### The standardization protocol of nCD64 and mHLA-DR showed excellent inter-rater reliability

**Figure 2:**
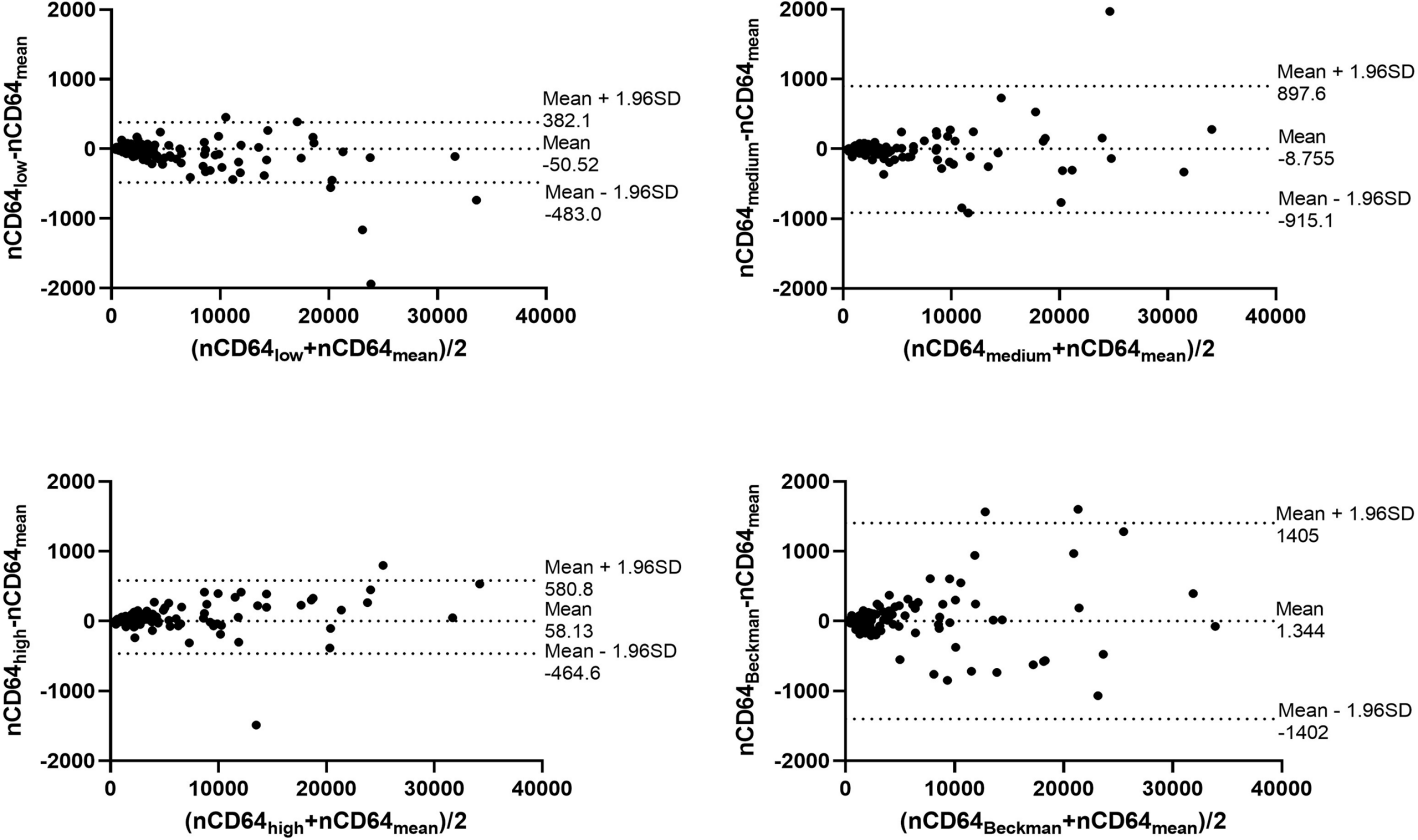
The Bland-Altman plot for the four MESFs of nCD64 under BD FACSCanto II with high/medium/high voltages and Beckman Coulter DxFlex with proper voltage. The agreement between the mean value of the four results and each result of nCD64 was assessed over the full range. The fixed range was defined as mean ± 1.96SD. nCD64, neutrophil CD64; SD, standard deviation; MESF, molecules of equivalent soluble fluorochrome.

**Figure 3:**
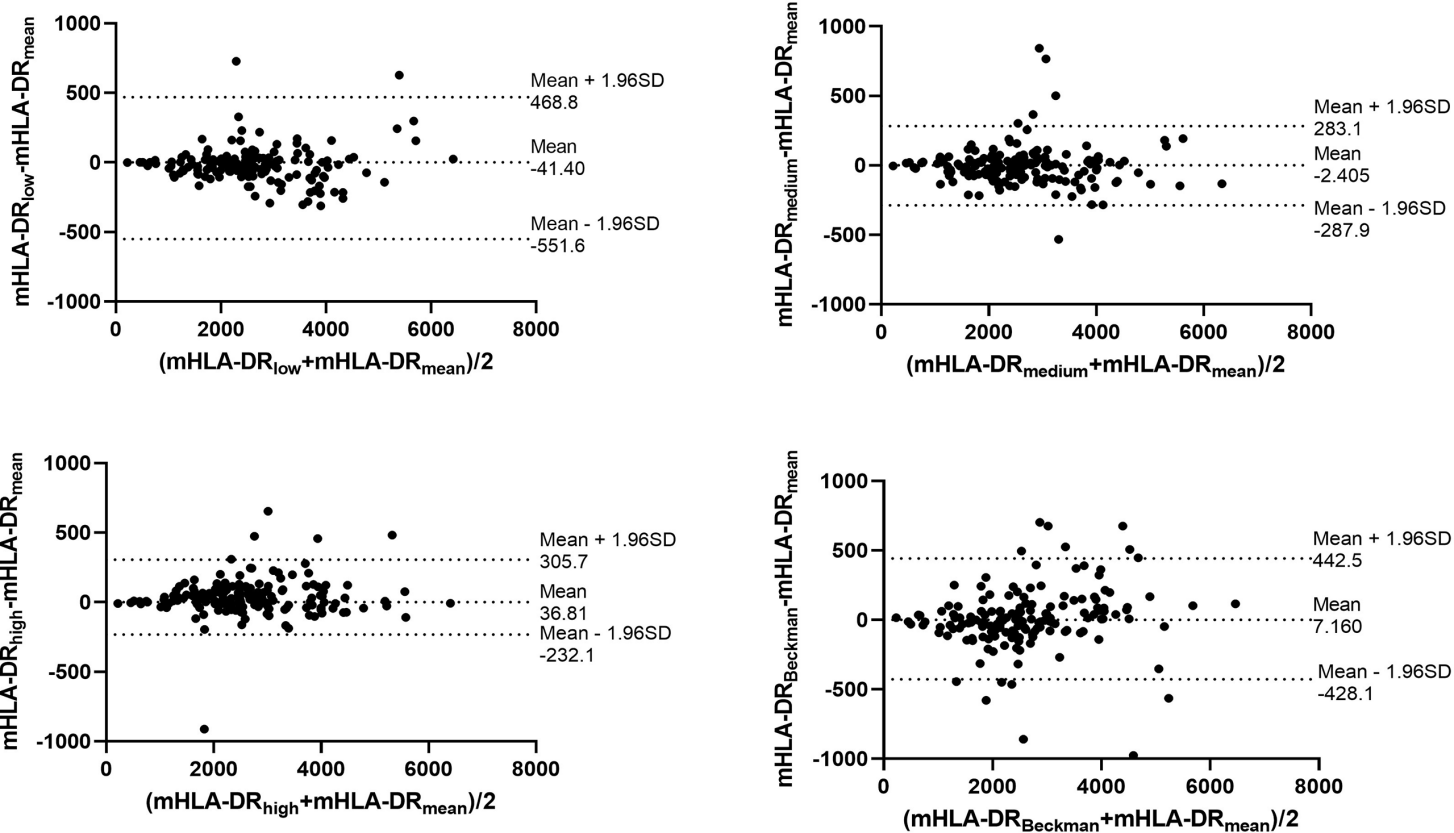
The Bland-Altman plot for the four MESFs of mHLA-DR under BD FACSCanto II with high/medium/high voltages and Beckman Coulter DxFlex with proper voltage. The agreement between the mean value of the four results and each result of mHLA-DR was assessed over the full range. The fixed range was defined as mean ± 1.96SD. mHLA-DR, monocyte HLA-DR; SD, standard deviation; MESF, molecules of equivalent soluble fluorochrome.

Through the calibrated beads with known number of PE molecules, the MFIs of nCD64 and mHLA-DR were converted to MESFs. The linear regression equations under the four settings were shown in the **Supplementary Figure 2**. Every sample was detected under the four settings as mentioned above, thus four MESF results for each sample were calculated. Then, the variability of the four results for each sample was evaluated using the ICC. Both the MESFs of nCD64 and mHLA-DR showed excellent reliability, with ICCs of 0.993 and 0.957, respectively (**supplementary Table 1**). Furthermore, the Bland-Altman test revealed a good agreement over the full range between the mean value of the four results and each result under the four settings. (**Figure 2** for nCD64 and **Figure 3** for mHLA-DR). To deal with the outliers which were defined as more than 3 SD in the Bland-Altman plots, the outliers were excluded and the mean value of the remaining results were used for further analysis. After removing the outliers, the Bland-Altman test showed better consistency (**Supplementary Figure 3** for nCD64 and **Supplementary Figure 4** for mHLA-DR). Overall, the standardization protocol of nCD64 and mHLA-DR showed excellent inter-rater reliability under different conditions.

### The infection KTRs had higher nCD64 but similar mHLA-DR compared with the stable KTRs

**Figure 4:**
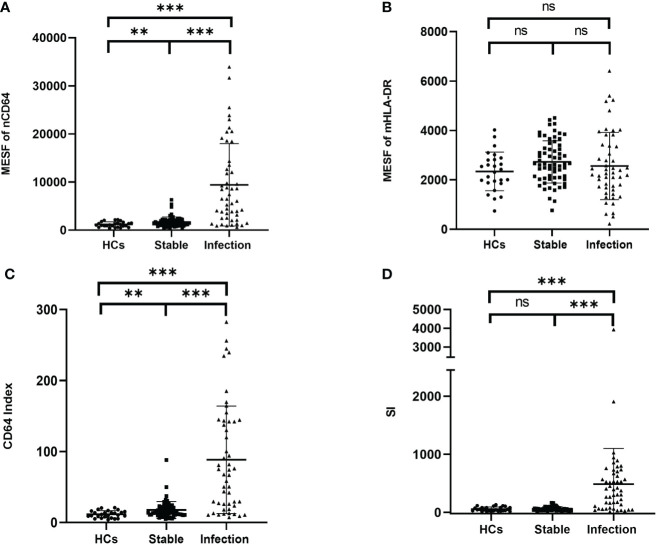
Comparison of the MESF of nCD64 **(A)**, MESF of mHLA-DR **(B)**, CD64 index **(C)** and SI **(D)** between HCs, stable KTRs and infection KTRs. Tested by the least significant difference test for back testing of multivariate ANOVA. *** means P < 0.001, and ** means P < 0.01. nCD64, neutrophil CD64; mHLA-DR, monocyte HLA-DR; HC, Healthy controls; KTRs, kidney transplant recipients; MESF, molecules of equivalent soluble fluorochrome; SI, Sepsis index; MESF, molecules of equivalent soluble fluorochrome. ns, no significance.

**Figure 5:**
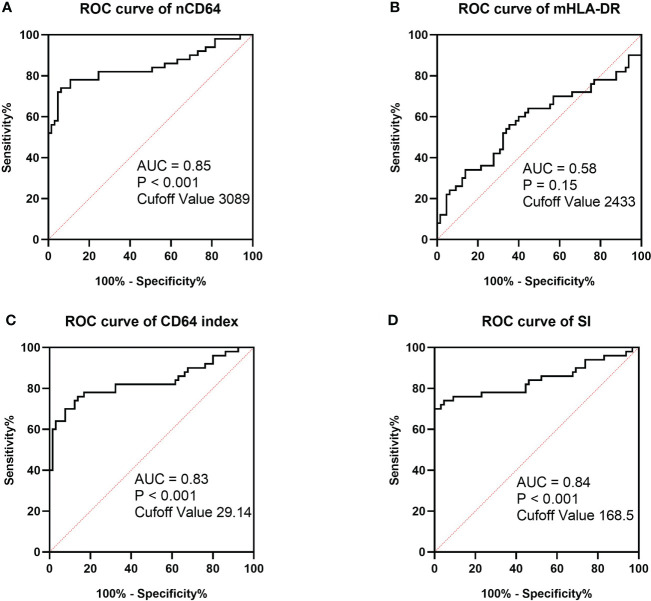
ROC curves of MESF of nCD64 **(A)**, MESF of mHLA-DR **(B)**, SI **(C)** and CD64 index **(D)** for diagnosis of infection in KTRs. The cut-off values were determined by the Youden index. ROC, Receiver operating characteristics; AUC, area under the curve; nCD64, neutrophil CD64; mHLA-DR, monocyte HLA-DR; KTRs, kidney transplant recipients; SI, Sepsis index; MESF, molecules of equivalent soluble fluorochrome.

To determine the performance of the immune monitoring panels in infection, the MESFs of nCD64 and mHLA-DR, CD64 index and SI were compared among the HCs, the infection group and the stable group (**Figure 4** and **Table 2**). For patients with multiple tests, the result of the first test was used for comparison. The stable group (1697.89 ± 1056.32) had slightly higher nCD64 than the HCs (1192.58 ± 537.61), but the nCD64 of the infection group (9424.08 ± 8574.58) was tremendously elevated compared with the other two groups. Surprisingly, the mHLA-DR showed no significant difference among the three groups (P = 0.273). Although the mHLA-DR of the infection group was a little lower than that of the stable group (2558.66 ± 1360.77 *vs*. 2728.62 ± 854.87), it showed no significant difference (P = 0.392). Due to the tremendous increase of nCD64, the CD64 index and SI of the infection group were also significantly higher than the other two groups. The ROC curves also showed that the nCD64, CD64 index and SI had high AUCs for identification of infection (AUCs 0.85, 0.83, 0.84, respectively, all P values less than 0.001), but the mHLA-DR showed no significant difference (P = 0.15, **Figure 5**).

**Table 2:** The expressions of nCD64 and mHLA-DR of the HCs and KTRs.

Parameters	All cases (n = 141)	HCs (n = 26)	KTRs			*P* value^#^
			Stable KTRs (n = 65)	Infection KTRs (n = 50)	*P* value^*^	
MESF of nCD64	4344.50 ± 6372.37	1192.58 ± 537.61	1697.89 ± 1056.32	9424.08 ± 8574.58	< 0.001	< 0.001
MESF of mHLA-DR	2596.92 ± 1054.52	2341.27 ± 781.75	2728.62 ± 854.87	2558.66 ± 1360.77	0.392	0.273
CD64 Index	41.58 ± 57.36	11.63 ± 4.97	17.60 ± 12.03	88.33 ± 75.69	< 0.001	< 0.001
SI	213.91 ± 417.41	58.86 ± 35.74	64.50 ± 32.69	488.76 ± 613.43	< 0.001	< 0.001

*Comparison between the stable KTRs and the infection KTRs.

^#^Comparison among the three groups.

Tested by the least significant difference test for back testing of multivariate ANOVA.

HCs, Healthy controls; KTRs, kidney transplant recipients, nCD64, neutrophil CD64; mHLA-DR, monocyte HLA-DR; MESF, molecules of equivalent soluble fluorochrome; SI, sepsis index.

For the TBNK panel, there were significant differences of the cell counts of CD3^+^ T cells, CD8^+^ T cells, CD4^+^ T cells, NK cells and B cells among the three groups (P ≤ 0.001). Compared with the stable group, the infection group was characterized by significantly lower cell counts of CD3^+^ T cells (1141.11 ± 537.85 *vs*. 628.52 ± 469.07, P < 0.001), CD8^+^ T cells (471.15 ± 242.78 *vs*. 284.68 ± 243.10, P < 0.001), CD4^+^ T cells (598.42 ± 322.91 *vs*. 301.36 ± 228.33, P < 0.001), NK cells (223.26 ± 204.71 *vs*. 123.40 ± 104.91, P < 0.001) and B cells (162.00 ± 113.84 *vs*. 67.17 ± 66.67, P < 0.001) (**Supplementary Table 2**). However, the percentages of each subset showed no significant difference.

### nCD64 helped distinguish different pathogenic pathogens of the infection

**Figure 6:**
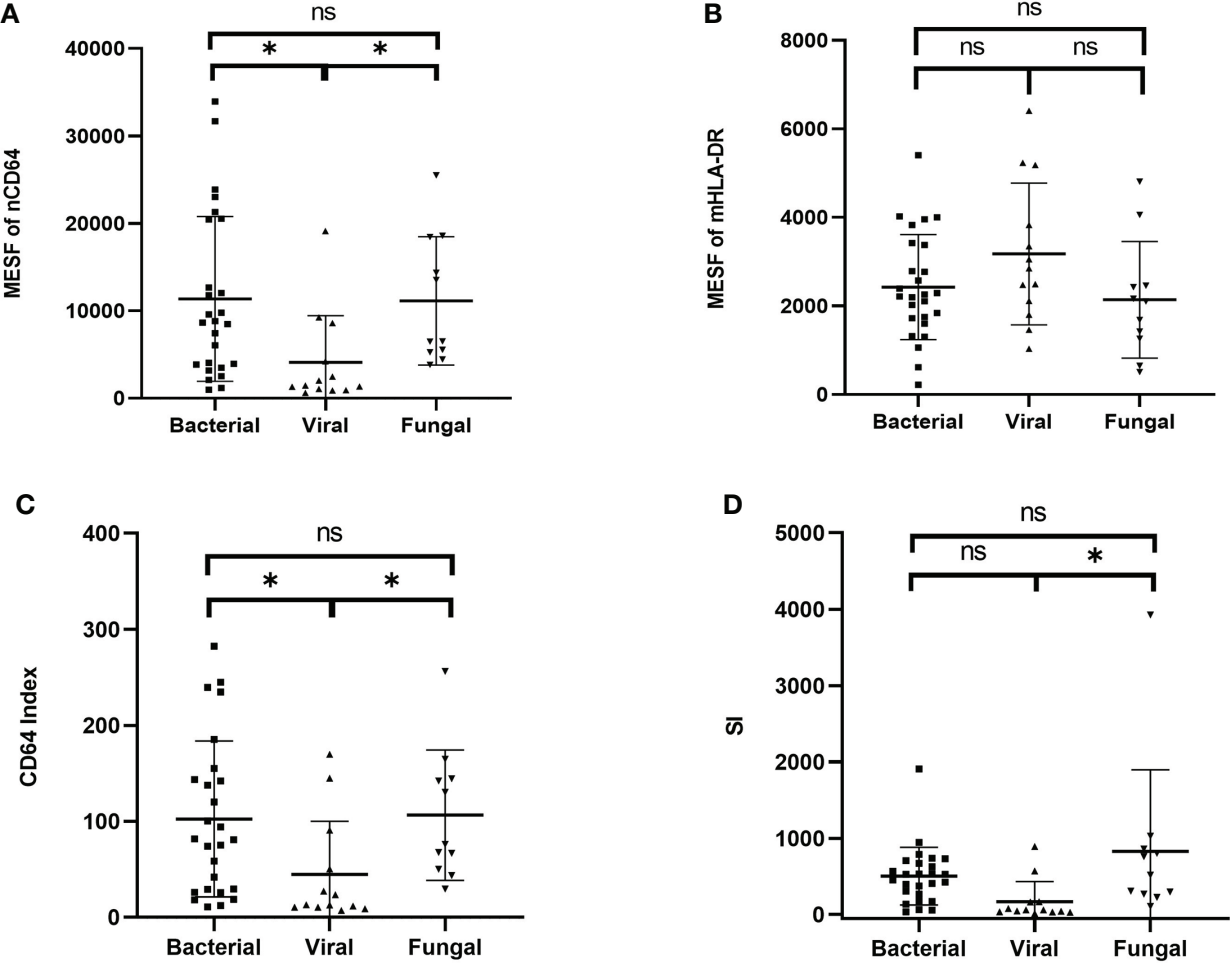
Comparison of the MESF of nCD64 **(A)**, MESF of mHLA-DR **(B)**, CD64 index **(C)** and SI **(D)** between the bacterial, the viral and the fungal infection subgroups. Tested by the least significant difference test for back testing of multivariate ANOVA. * means P < 0.05. nCD64, neutrophil CD64; mHLA-DR, monocyte HLA-DR; HC, Healthy controls; KTRs, kidney transplant recipients; MESF, molecules of equivalent soluble fluorochrome; ns, no significance.

According to the different pathogenic pathogens, the infection group (n = 50) were further stratified into the bacterial (n = 26), the viral (n = 13) and the fungal (n = 11) infection subgroups. MESFs of nCD64 and mHLA-DR, CD64 index and SI were compared among the infection subgroups according to the pathogenic pathogens. The expression of nCD64 was significantly higher in the bacterial and fungal infection subgroups than that in the viral infection subgroup. Although the mHLA-DR in the viral infection subgroup slightly increased, there was no statistical difference (**Table 3**). For the comparisons between every two subgroups, the bacterial and the fungal infection subgroups showed similar characteristics of nCD64, CD64 index and SI, while these parameters were lower in the viral infection subgroup (**Figure 6**).

**Table 3:** The expressions of nCD64 and mHLA-DR of infection KTRs with different pathogens.

Parameters	Bacterial (n = 26)	Viral (n = 13)	Fungal (n = 11)	*P* value^#^
MESF of nCD64	11361.46 ± 9428.95	4099.15 ± 5347.64	11137.91 ± 7337.20	0.03
MESF of mHLA-DR	2426.73 ± 1187.87	3176.31 ± 1601.28	2140.55 ± 1317.32	0.14
CD64 index	102.42 ± 81.11	44.75 ± 55.29	106.51 ± 67.89	0.051
SI	504.29 ± 376.84	170.73 ± 262.07	827.91 ± 1071.35	0.029

^#^Comparison among the three groups.

Tested by the least significant difference test for back testing of multivariate ANOVA.

KTRs, kidney transplant recipients; nCD64, neutrophil CD64; mHLA-DR, monocyte HLA-DR; MESF, molecules of equivalent soluble fluorochrome; SI, sepsis index.

### mHLA-DR and SI identified sepsis in KTRs with infection

**Figure 7:**
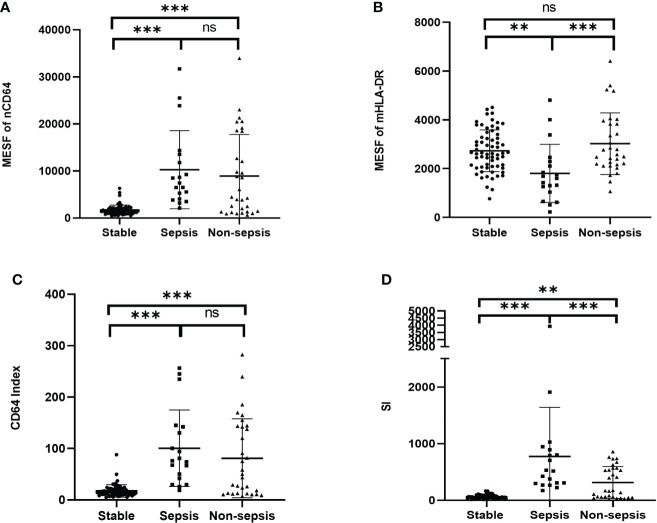
Comparison of the MESF of nCD64 **(A)**, MESF of mHLA-DR **(B)**, CD64 index **(C)** and SI **(D)** between the stable, the sepsis and the non-sepsis groups. Tested by the least significant difference test for back testing of multivariate ANOVA. *** means P < 0.001 and ** means P < 0.01. nCD64, neutrophil CD64; mHLA-DR, monocyte HLA-DR; HC, Healthy controls; KTRs, kidney transplant recipients; MESF, molecules of equivalent soluble fluorochrome; SI, Sepsis index. ns, no significance.

**Figure 8:**
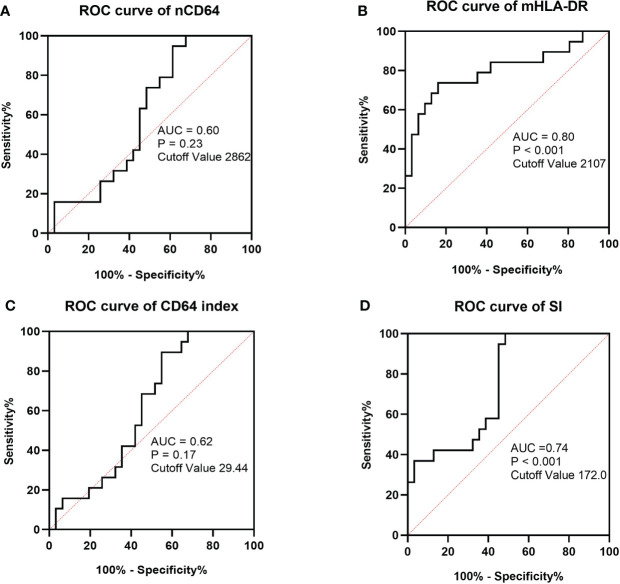
ROC curves of MESF of nCD64 **(A)**, MESF of mHLA-DR **(B)**, SI **(C)** and CD64 index **(D)** for diagnosis of sepsis in KTRs with infection. The cut-off values were determined by the Youden index. ROC, Receiver operating characteristics; AUC, area under the curve; nCD64, neutrophil CD64; mHLA-DR, monocyte HLA-DR; KTRs, kidney transplant recipients; SI, Sepsis index.

Sepsis was life-threatening organ dysfunction caused by a dysregulated host response to infection. KTRs with sepsis had a much higher mortality rate than the non-sepsis patients. Because the stable KTRs might maintain a relatively poorer renal function than the HCs, the SOFA score relating to the renal function was adjusted as mentioned in the methods. Although the MESFs of nCD64 and mHLA-DR, CD64 index and SI showed significant differences among the stable, the sepsis and the non-sepsis groups, nCD64 and CD64 index could not distinguish between sepsis and non-sepsis patients (**Table 4** and **Figure 7**). The sepsis patients had much lower mHLA-DR than the non-sepsis and the stable groups, but the nCD64 of the sepsis and the non-sepsis patients was close (both of them were higher than the stable group). Therefore, the sepsis group had the highest SI among the three groups. The non-sepsis group had the second highest SI, although the mHLA-DR showed no significant difference between the non-sepsis and the stable groups. The ROC curves confirmed that mHLA-DR and SI had good performance to identify sepsis in KTRs, with AUCs of 0.80 and 0.74, respectively (**Figure 8**).

**Table 4:** The expressions of nCD64 and mHLA-DR of infection KTRs with sepsis.

Parameters	Stable KTRs (n = 65)	Infection KTRs (n = 50)			*P* value^#^
		Sepsis (n = 19)	Non-sepsis (n = 31)	*P* value^*^	
MESF of nCD64	1697.89 ± 1056.32	10265.47 ± 8293.09	8908.39 ± 8837.41	0.416	< 0.001
MESF of mHLA-DR	2728.62 ± 854.87	1803.47 ± 1192.66	3021.52 ± 1260.32	< 0.001	< 0.001
CD64 index	17.60 ± 12.03	100.47 ± 74.46	80.98 ± 76.68	0.186	< 0.001
SI	64.50 ± 32.69	773.96 ± 868.31	313.96 ± 281.34	< 0.001	< 0.001

*Comparison between the sepsis patients and non-sepsis patients.

^#^Comparison among the three groups.

Tested by the least significant difference test for back testing of multivariate ANOVA.

KTRs, kidney transplant recipients; nCD64, neutrophil CD64; mHLA-DR, monocyte HLA-DR; MESF, molecules of equivalent soluble fluorochrome; SI, sepsis index.

**Figure 9:**
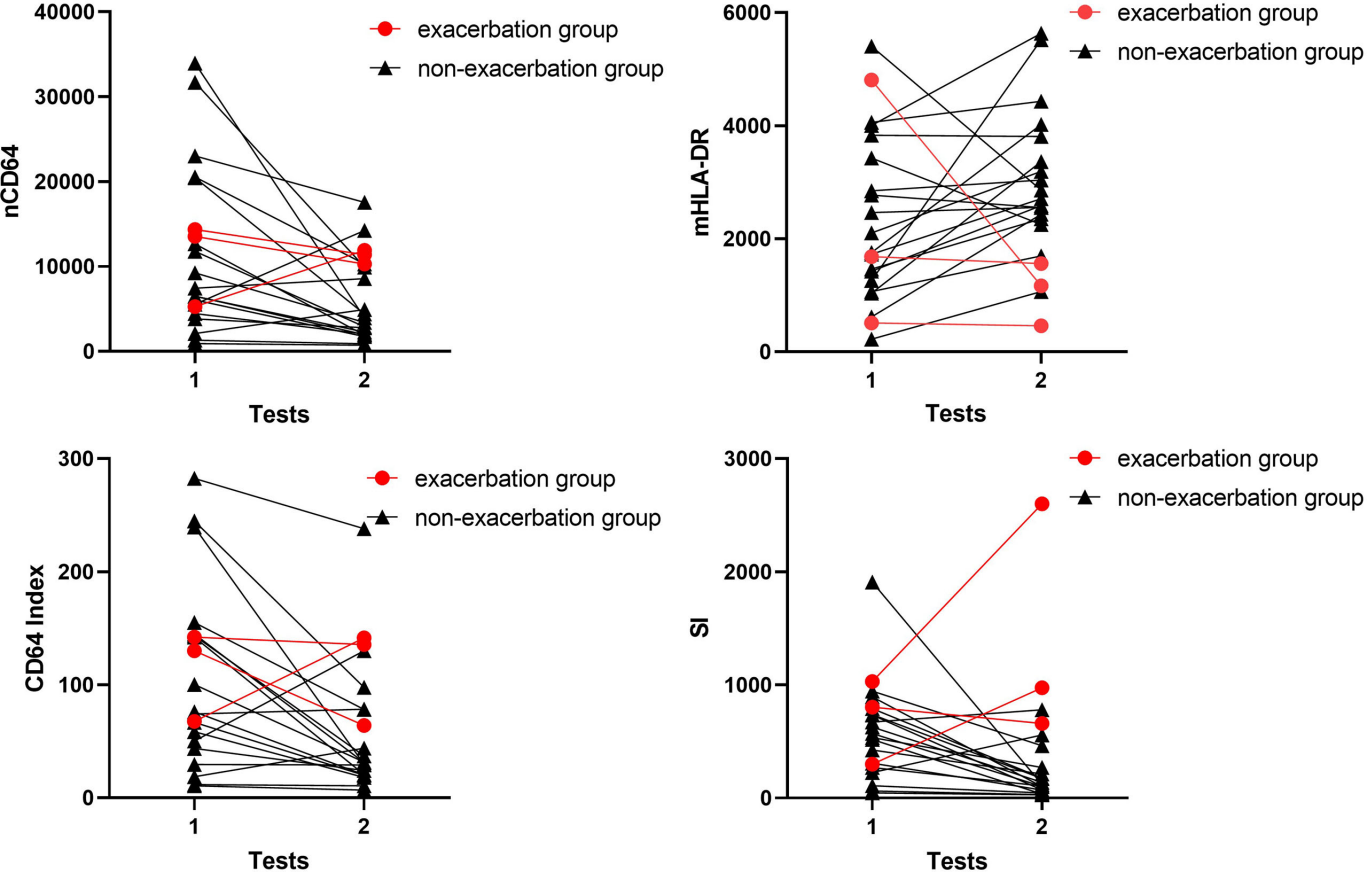
Longitudinal results of the immune monitoring panels in infection KTRs. The changes of MESFs of nCD64 and mHLA-DR, CD64 index and SI were shown for each patient. The exacerbation group was defined as ΔSOFA > 0 and the non-exacerbation group was defined as ΔSOFA ≤ 0. nCD64, neutrophil CD64; mHLA-DR, monocyte HLA-DR; KTRs, kidney transplant recipients; MESF, molecules of equivalent soluble fluorochrome; SI, Sepsis index; ΔSOFA, change of Sequential Organ Failure Assessment score.

As shown in **Figure 1**, 21 infection patients received the immune monitoring panels twice at the interval of approximately 1 week. These patients were also assessed by the SOFA score twice when they received the immune monitoring tests. According to the change of SOFA scores, which presented the severity of the infection, the patients were stratified into the exacerbation group (ΔSOFA > 0) and the non-exacerbation group (ΔSOFA ≤ 0). The exacerbation group had a sharp decline of mHLA-DR but a significant increase of SI, which suggested that dynamic changes of mHLA-DR and SI were related to the prognosis of the infection (**Table 5** and **Figure 9**). In addition, the dynamic change of the nCD64 and CD64 index showed no significant relation to the prognosis.

**Table 5:** The dynamic changes of nCD64 and mHLA-DR expression related to the prognosis of the infection KTRs.

Parameters	Infection KTRs (n = 21)	Exacerbation group (n = 3)	Non-exacerbation group (n = 18)	*P* value*
ΔnCD64	-5387.71 ± 8938.28	143.67 ± 5631.89	-6309.61 ± 9163.02	0.26
ΔmHLA-DR	513.29 ± 1671.85	-1273.33 ± 2049.93	811.06 ± 1461.78	0.042
ΔSI	-228.19 ± 635.51	701.67 ± 857.79	-383.17 ± 458.87	0.003
ΔCD64 index	-45.10 ± 70.38	0.33 ± 70.29	-52.67 ± 69.44	0.24

*Comparison between the exacerbation group and non-exacerbation group.

Tested by Student’s t test or Welch’s t test.

The exacerbation group was defined as ΔSOFA > 0 and the non-exacerbation group was defined as ΔSOFA ≤ 0. Δ means the results of the second test minus that of the first test.

KTRs, kidney transplant recipients; nCD64, neutrophil CD64; mHLA-DR, monocyte HLA-DR; MESF, molecules of equivalent soluble fluorochrome; SI, sepsis index.

### nCD64 and B cell counts were independent risk factors of infection

To identify the risk factors of infection in KTRs, logistic analysis was performed with nCD64, mHLA-DR and parameters of the TBNK panel. Among them, nCD64 and mHLA-DR were transformed into binary variables with the cut-off values determined by the Youden index. As shown in **Table 6**, univariate logistic analysis revealed that nCD64 > 3089 (unadjusted odds ratio (OR) 43.404; 95% confidence interval (CI) 13.168 – 143.065; P < 0.001), mHLA-DR > 2433 (unadjusted OR 0.430; 95% CI 0.202 – 0.916; P = 0.029), the cell counts of CD3^+^ T cells (unadjusted OR 0.998; 95% CI 0.997 – 0.999; P < 0.001), the cell counts of CD8^+^ T cells (unadjusted OR 0.996; 95% CI 0.995 – 0.998; P < 0.001), the cell counts of CD4^+^ T cells (unadjusted OR 0.996; 95% CI 0.994 – 0.998; P < 0.001), the cell counts of NK cells (unadjusted OR 0.995; 95% CI 0.992 – 0.998; P < 0.001), and the cell counts of B cells (unadjusted OR 0.986; 95% CI 0.979 – 0.993; P < 0.001) were significantly associated with infection. Multivariate analysis further revealed that nCD64 > 3089 (unadjusted OR 49.378; 95% CI 11.015 – 221.347; P < 0.001) and the cell counts of B cells (unadjusted OR 0.989; 95% CI 0.979 – 0.998; P = 0.022) were independent risk factors for the infection in KTRs.

**Table 6:** Univariate and multivariate odds ratios for infection diagnosis among KTRs.

Parameters	Univariate analysis		Multivariate analysis	
	OR (95% CI)	*P* value	OR (95% CI)	*P* value
MESF of nCD64 > 3089	43.404 (13.168 – 143.065)	< 0.001	49.378 (11.015 - 221.347)	< 0.001
MESF of mHLA-DR > 2433	0.430 (0.202 – 0.916)	0.029	0.422 (0.130 – 1.372)	0.151
CD3^+^ T cells/TBNK, mean ± SD (%)	0.991 (0.954 - 1.029)	0.644		
CD3^+^ T cells, n ± SD (cells/μl)	0.998 (0.997 - 0.999)	< 0.001	0.996 (0.983 - 1.009)	0.548
CD8^+^ T cells/TBNK, mean ± SD (%)	1.021 (0.977 - 1.067)	0.352		
CD8^+^ T cells, n ± SD (cells/μl)	0.996 (0.995 - 0.998)	< 0.001	1.008 (0.993 - 1.022)	0.284
CD4^+^ T cells/TBNK, mean ± SD (%)	0.976 (0.943 - 1.011)	0.176		
CD4^+^ T cells, n ± SD (cells/μl)	0.996 (0.994 - 0.998)	< 0.001	1.002 (0.989 - 1.014)	0.811
NK cells/TBNK, mean ± SD (%)	1.031 (0.984 - 1.079)	0.202		
NK cells, n ± SD (cells/μl)	0.995 (0.992 - 0.998)	0.005	1.001 (0.997 - 1.005)	0.600
B cells/TBNK, mean ± SD (%)	0971 (0.913 - 1.033)	0.359		
B cells, n ± SD (cells/μl)	0.986 (0.979 - 0.993)	< 0.001	0.989 (0.979 - 0.998)	0.022
CD4/CD8 ratio, mean ± SD	0.719 (0.413 - 1.251)	0.243		

OR, odds ratio; TBNK, T, B, and NK cells; NK cells, natural killer cells; nCD64, neutrophil CD64; mHLA-DR, monocyte HLA-DR; MESF, molecules of equivalent soluble fluorochrome.

## Discussion

In this study, we successfully established a standardization protocol of nCD64 and mHLA-DR and assessed their performance in KTRs with infection. nCD64 had the best diagnostic performance in KTRs with infection, and mHLA-DR distinguished sepsis and the dynamic change of mHLA-DR correlated with prognosis. mHLA-DR and nCD64 provided direct information of the immune status KTRs, and standardization measurement contributed to promotion and application of these parameters.

Infection is one of the most common but serious complications following KTx. Previous study using national data of KTRs indicated that the cumulative incidence of post-transplant infection was as high as 36.9% at 3 months, 53.7% at 1 year, 69.6% at 3 years, and 78.0% at 5 years (2). The most common post-transplant infections were urinary tract infection (46.8%) and pneumonia (28.2%). Because the optimal range balancing rejection and infection of the immunosuppressive drugs for KTRs is narrow, roughly adjusting the immunosuppressive drugs without precise immunological assessment during infection is likely to increase the risk of rejection or aggravate infection. Therefore, reliable immune biomarkers are needed to accurately assess immune status for KTRs with infection (19).

CD64 is constitutively expressed on monocytes, eosinophils, and neutrophils, but negatively expressed on lymphocytes. In a resting state, the expression of CD64 on monocytes is relatively high, but on neutrophils the expression is relatively low. However, a rapid 10-fold increase of CD64 expression is on neutrophils in a short period of time (4 – 6 hours) following an inflammatory response or pro-inflammatory cytokine stimulation, which plays an instrumental role in the immune response to infection (20). Meanwhile, the increase of CD64 expression on monocytes is limited. Therefore, nCD64 and CD64 index are effective and sensitive biomarkers for inflammation in the early stage. In our study, the stable KTRs had slightly higher nCD64 and CD64 index than the HCs, suggesting that mild inflammation response persisted in the stable KTRs. When the KTRs suffered from infection, both nCD64 and CD64 index rose sharply even if the KTRs received immunosuppressive drugs and were under immunosuppression. Therefore, nCD64 and CD64 index were still available biomarkers of infection for KTRs.

Different pathogenic pathogens of the infection showed different characteristics in the change of nCD64. It was reported that nCD64 expression was elevated in bacterial infections while it was normal in viral infections, which had potential for accurately distinguishing bacterial from COVID-19 or other viral infections in the emergency department (21). Pander G et al. indicated that quantitative detection of nCD64 in patients with severe alcoholic hepatitis could more accurately identify systemic bacterial infections and inflammation compared to other inflammatory markers (22). Consistently, our study also showed that KTRs with bacterial infections had significantly higher nCD64 and CD64 index than those with viral infections. The fungal infections, most of which were caused by Pneumocystis *jirovecii* in our study, also showed much higher nCD64 and CD64 index than the viral infections. When pathogens entered the human body, the innate immune system was activated by inducing cytokines, such as interferon (IFN), which was primarily involved in host defense against invading pathogens. IFNs were classified into three distinct types including type I, type II (gamma), and type III. *In vitro* and *in vivo* experiments demonstrated that IFN-γ intensely stimulated the expression of nCD64, but little effect was seen with type I and type III IFNs (23, 24). Upon bacterial infection, the activation of the immune system released pro-inflammatory cytokines including IFN-γ, which strongly induced the expression of nCD64 (24). Moreover, bacterial components such as lipopolysaccharide (LPS) could also induce CD64 expression on the surface of neutrophils (24). Similarly, the fungal infection, especially caused by Pneumocystis *jirovecii*, could also induce the secretion of IFN-γ, thus significantly upregulating the expression of nCD64 (25–27). In contrast, viral infection mainly induced the secretion of IFN type I, which slightly upregulated nCD64 (24). Yet it was worth noting that infections in KTRs were commonly mixed infections, thus nCD64 could only suggest but not confirm the pathogens of infection.

Currently, some researches showed that nCD64 expression performed good diagnostic efficacy in bacterial infections and sepsis (22). In a prospective observational study, it concluded that serial measurement of nCD64 expression could facilitate sepsis diagnosis and monitor the clinical course in critically ill patients (17). However, a meta-analysis indicated that nCD64 expression was a helpful marker but not sufficient for early diagnosis of sepsis in critically ill adult patients (28), which yielded conflicting results. In our study, the sepsis group and non-sepsis group had close nCD64 and CD64 index, although both of them were much higher than the stable group and HCs. Therefore, our data suggested that nCD64 alone was not enough to distinguish sepsis in KTRs with infection.

mHLA-DR expression is the most recognized immune indicator to assess the degree of immunosuppression in critically ill patients (29). Under the condition of sepsis, monocytes with decreased HLA-DR expression exhibit an impaired capacity to mount a proinflammatory reaction upon a secondary bacterial challenge and impairment in antigen presentation capacity (30). A wide array of studies confirmed that decreased mHLA-DR expression was a predictor for sepsis or septic complications and correlated with prognosis (30). A multi-centre cohort study showed that lower mHLA-DR expression with increased neutrophil CD24 and neutrophil CD279 best predicted the clinical deterioration to sepsis (31). Leijte et al. also indicated that dynamic declination of mHLA-DR over time was associated with adverse clinical outcomes of septic shock, but mHLA-DR expression exhibited no significant association with causative pathogens (32). In KTRs, the long-term administration of immunosuppressive drugs indeed leads to persist immunosuppression, but the classic triple immunosuppressive regimens mainly suppress the adaptive immunity. Therefore, stable KTRs and even KTRs with infection showed no significant decrease of mHLA-DR in this study. Only KTRs with sepsis, which indicated that patients had severe infection and were severely immunosuppressed, had decreased expression of mHLA-DR. It was shown in our study that mHLA-DR exhibited the best performance to distinguish sepsis among KTRs with infection. The SI which combined mHLA-DR and nCD64 also contributed to identifying patients with sepsis. In addition, the persistent decline of mHLA-DR was correlated with aggravation of infection.

In our study, the cell count of B cells was identified as an independent risk factor for KTRs with infection in multivariate logistic regression analysis. Although B cells compromised a relatively small percentage of lymphocytes, they played a critical role in adaptive immunity to fight against infection (33). Patients with decreased B cells were predisposed to infection. In our previous study, KTRs with pneumonia were characterized with significantly lower cell count of B cells, which was in accordance with the results in this research (16). Rituximab, a monoclonal antibody that depleted B cells, was reported to cause hypogammaglobulinemia and increased risk of severe infection (34). For KTRs, the use of rituximab after transplantation was also associated with higher risk of infectious disease and lower survival rate (35). Therefore, we should pay more attention to the B cells as a biomarker for immune monitoring.

In previous studies, nCD64 and mHLA-DR were quantified by the percentage of cells positively expressed these markers or the MFI values. Indeed, both CD64 and HLA-DR were continuously expressed on cells, which meant that it was technically difficult to determine the cut-off values for gating the positive cells. In addition, different gating strategies notably affected the results. For nCD64, almost all neutrophils positively expressed CD64 when patients suffered from infection, thus making it meaningless for comparison. The value of MFI was indeed quantified and widely used. However, MFI was a relative value, which was also determined by the instrument settings (such as voltage of the photomultipliers, optical filters, etc.) and antibodies selected. Therefore, the results of nCD64 and mHLA-DR quantified by MFIs varied in different studies, and it was meaningless for comparison between different studies. In this study, a standardization protocol of nCD64 and mHLA-DR was established. The MFI was converted into MESF by the calibrated beads with known number of PE molecules. It proved good reliability under different settings and instruments, making it possible for lab-to-lab comparison. In this protocol, some details should be noted. There should be no compensation for the channel chosen for standardization in the multicolor flow cytometry panel. The voltage for the channel should be fixed and the routine calibration of the flow cytometer should be performed.

There were some limitations in this study. The sample size was relatively limited, and the patients were recruited from a single institution. nCD64 and mHLA-DR were detected at only two points over two weeks, and some patients did not complete all tests because they were discharged. Due to the timely treatment, KTRs with infection had a relatively good prognosis in this study. Therefore, the prognostic value of nCD64 and mHLA-DR for infection needed further research.

In conclusion, we established a standardization protocol for detection of nCD64 and mHLA-DR, making it available for the lab-to-lab comparison and widespread application. nCD64 and mHLA-DR had good diagnostic performance in KTRs with infection and sepsis, respectively, which could be promising indicators for immune monitoring of KTRs and contributed to individualized treatment.

## Data availability statement

The raw data supporting the conclusions of this article will be made available by the authors, without undue reservation.

## Ethics statement

The studies involving human participants were reviewed and approved by The Ethics committee of Third Xiangya Hospital, Central South University. The patients/participants provided their written informed consent to participate in this study. Written informed consent was obtained from the individual(s) for the publication of any potentially identifiable images or data included in this article.

## Author contributions

BP and YM: critical analysis, interpretation of the data, and drafting of the manuscript. MY and QZ: sample processing and cohort management. MY and JL: clinical data collection and sample acquisition. PZ, HL, and KC: guidance of experiment and technical support. BP and YM: conceived and designed the study. All authors contributed to the article and approved the submitted version.

## Funding

This study was supported by the National Natural Science Foundation of China (81771722) and the Hunan Provincial Natural Science Foundation of China (2020JJ5863). The funders had no role in study design, data collection, analysis and interpretation, writing and submission of the manuscript.

## Acknowledgments

We thank all the individuals for their participation in this study.

## Conflict of interest

The authors declare that the research was conducted in the absence of any commercial or financial relationships that could be construed as a potential conflict of interest.

## Publisher’s note

All claims expressed in this article are solely those of the authors and do not necessarily represent those of their affiliated organizations, or those of the publisher, the editors and the reviewers. Any product that may be evaluated in this article, or claim that may be made by its manufacturer, is not guaranteed or endorsed by the publisher.
