# Supplementary material for: Standardization of neutrophil CD64 and monocyte HLA-DR measurement and its application in immune monitoring in kidney transplantation
Source: Front Immunol. 2022 Nov 23;13:1063957. doi: 10.3389/fimmu.2022.1063957 (PMC9727265; doi:10.3389/fimmu.2022.1063957)
Supplement: Supplementary file 1 [file DataSheet_1.doc]

Supplementary Material

Supplementary Table 1 Inter-rater reliability of the MESF of nCD64 and mHLA-DR

| Parameters | Intraclass correlation | 95% CI | F Test | | | |
| --- | --- | --- | --- | --- | --- | --- |
|  |  |  | Value | df1 | df2 | Sig |
| MESF of nCD64 | 0.993 | 0.991 - 0.994 | 543.293 | 161 | 483 | < 0.001 |
| MESF of mHLA-DR | 0.957 | 0.945 - 0.967 | 91.018 | 161 | 483 | < 0.001 |

The reliability of the MESF of nCD64 and mHLA-DR for four results with BD FACSCanto II with high/medium/high voltage and Beckman Coulter DxFlex with proper voltage was was calculated by intraclass correlation coefficient (ICC). nCD64, neutrophil CD64; mHLA-DR, monocyte HLA-DR; MESF, molecules of equivalent soluble fluorochrome.

Supplementary Table 2 The regular immune monitoring panel of the HCs and KTRs

| Parameters | All cases (n = 141) | HCs (n = 26) | KTRs | |  | *P* value^#^ |
| --- | --- | --- | --- | --- | --- | --- |
|  |  |  | Stable KTRs (n = 65) | Infection KTRs (n = 50) | *P* value^*^ |  |
| CD3^+^ T cells/TBNK, mean ± SD (%) | 73.41 ± 9.30 | 71.91 ± 6.65 | 74.12 ± 8.60 | 73.27 ± 11.23 | 0.960 | 0.591 |
| CD3^+^ T cells, n ± SD (cells/μl) | 954.26 ± 536.37 | 1113.54 ± 331.53 | 1141.11 ± 537.85 | 628.52 ± 469.07 | < 0.001 | < 0.001 |
| CD8^+^ T cells/TBNK, mean ± SD (%) | 31.25 ± 8.01 | 29.39 ± 4.88 | 31.03 ± 8.11 | 32.52 ± 9.04 | 0.740 | 0.260 |
| CD8^+^ T cells, n ± SD (cells/μl) | 401.78 ± 242.21 | 453.54 ± 138.13 | 471.15 ± 242.78 | 284.68 ± 243.10 | < 0.001 | < 0.001 |
| CD4^+^ T cells/TBNK, mean ± SD (%) | 37.32 ± 10.24 | 37.18 ± 5.81 | 38.57 ± 8.95 | 35.76 ± 13.15 | 0.483 | 0.345 |
| CD4^+^ T cells, n ± SD (cells/μl) | 488.92 ± 303.40 | 575.88 ± 192.25 | 598.42 ± 322.91 | 301.36 ± 228.33 | < 0.001 | < 0.001 |
| NK cells/TBNK, mean ± SD (%) | 14.58 ± 7.68 | 15.51 ± 5.44 | 13.52 ± 7.83 | 15.47 ± 8.42 | 0.499 | 0.319 |
| NK cells, n ± SD (cells/μl) | 191.87 ± 167.34 | 245.04 ± 114.37 | 223.26 ± 204.71 | 123.40 ± 104.91 | 0.003 | 0.001 |
| B cells/TBNK, mean ± SD (%) | 10.25 ± 5.85 | 11.43 ± 3.86 | 10.45 ± 5.68 | 9.37 ± 6.82 | 0.752 | 0.327 |
| B cells, n ± SD (cells/μl) | 132.94 ± 110.41 | 186.77 ± 111.87 | 162.00 ± 113.84 | 67.17 ± 66.67 | < 0.001 | < 0.001 |
| CD4/CD8 ratio, mean ± SD | 1.33 ± 0.67 | 1.31 ± 0.37 | 1.40 ± 0.69 | 1.24 ± 0.76 | 0.570 | 0.442 |

* Comparison between the stable KTRs and the infection KTRs.

# Comparison among the three groups.

Tested by the least significant difference test for back testing of multivariate ANOVA. HCs, Healthy controls; KTRs, kidney transplant recipients.SD, standard deviation.


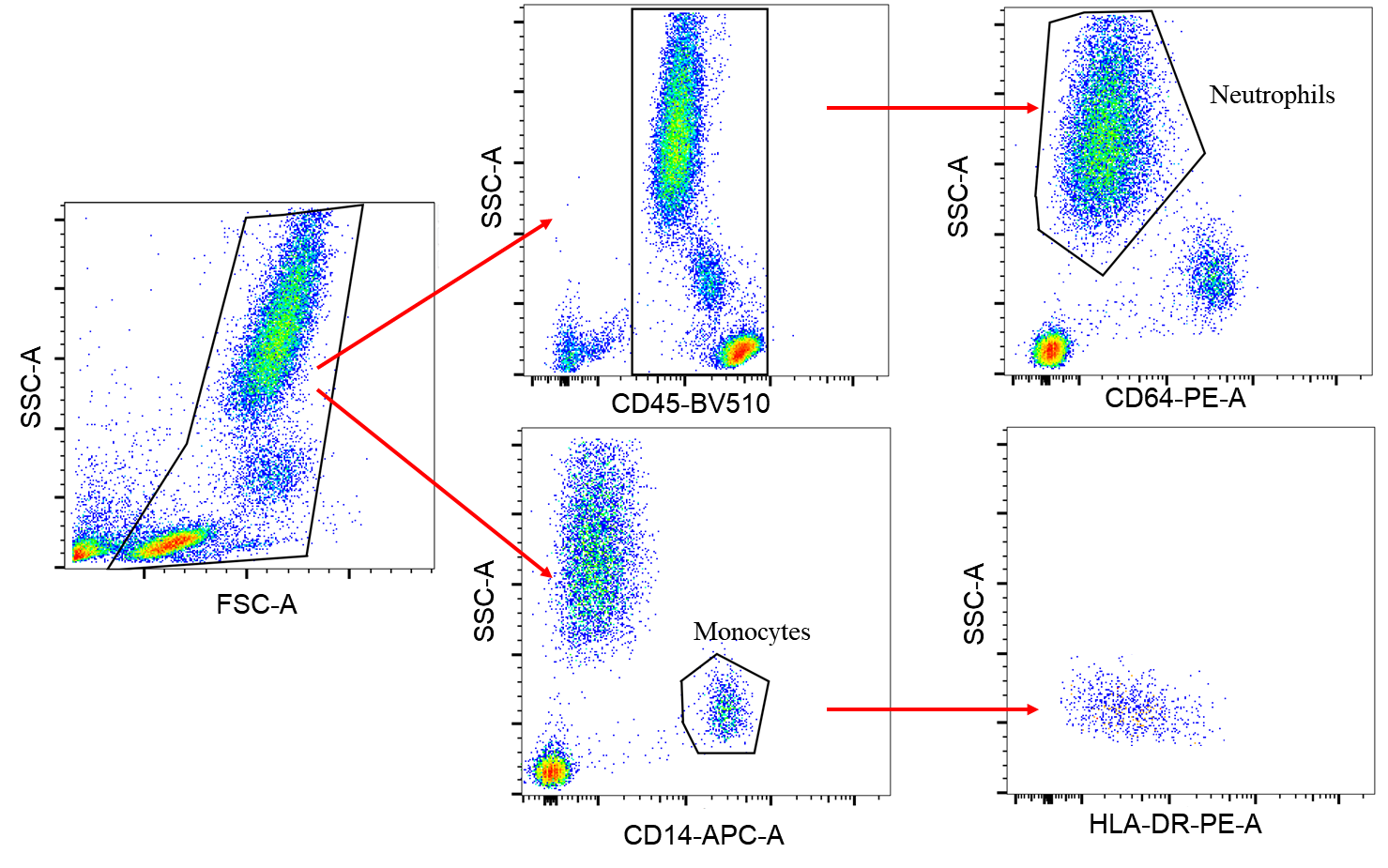


Supplementary Figure 1 Representative dot plots showing the gating strategy for nCD64 and mHLA-DR panels. After gating singlets, neutrophls were gated based on SSC/CD64 bivariate dot plot from CD45^+^ white blood cells, and monocytes were gated based on SSC/CD14 bivariate dot plot. nCD64, neutrophil CD64; mHLA-DR, monocyte HLA-DR.


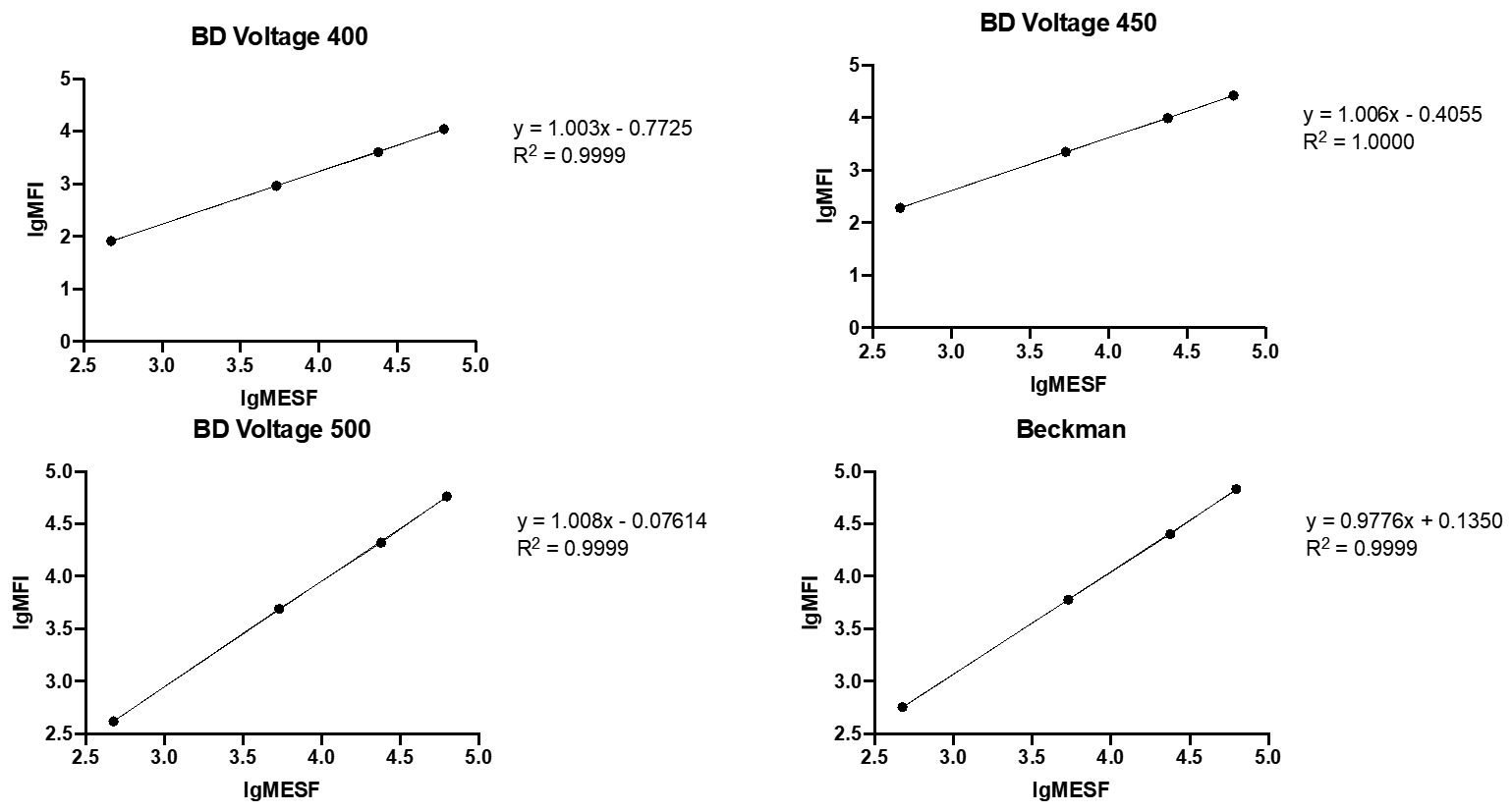


Supplementary Figure 2 The linear regression equations under the four settings for nCD64 and mHLA-DR. On the flow cytometer BD FACSCanto II, the voltage of PE channel was set at three levels. On the flow cytometer Beckman Coulter DxFlex, the voltage of PE channel was fixed. The linear regression of Log_10_ MFI against Log_10_ PE molecules per bead (namely the MESF value) was using the equation y = mx + c. nCD64, neutrophil CD64; mHLA-DR, monocyte HLA-DR; PE, Phycoerythrin; MFI, mean fluorescence intensities; MESF, molecules of equivalent soluble fluorochrome.


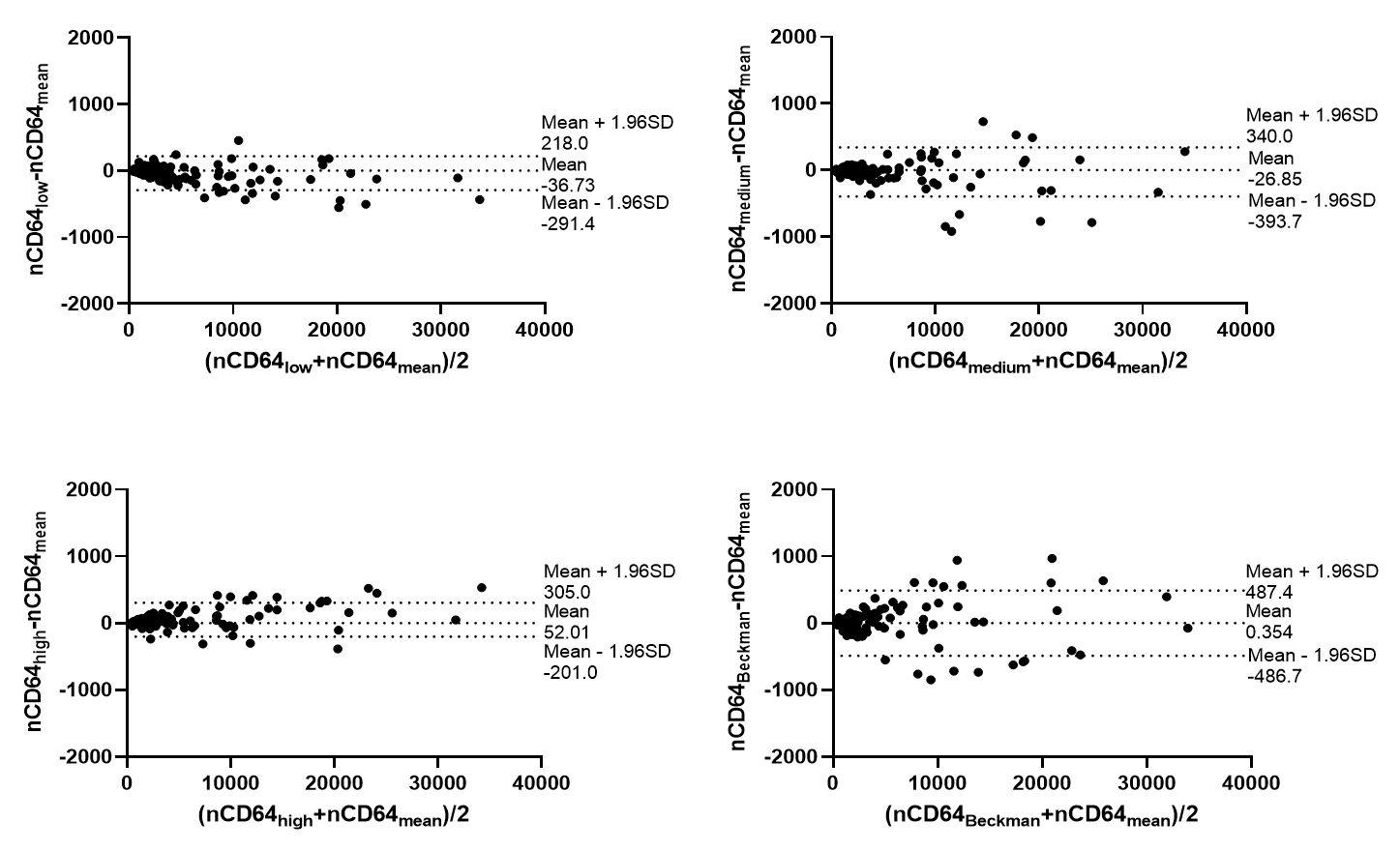


Supplementary Figure 3 The Bland-Altman plot for the four MESFs of nCD64 under BD FACSCanto II with high/medium/high voltages and Beckman Coulter DxFlex with proper voltage after removing the outliers. The agreement between the mean value of the four results and each result of nCD64 was assessed over the full range. The fixed range was defined as mean ± 1.96SD. nCD64, neutrophil CD64; SD, standard deviation; MESF, molecules of equivalent soluble fluorochrome.


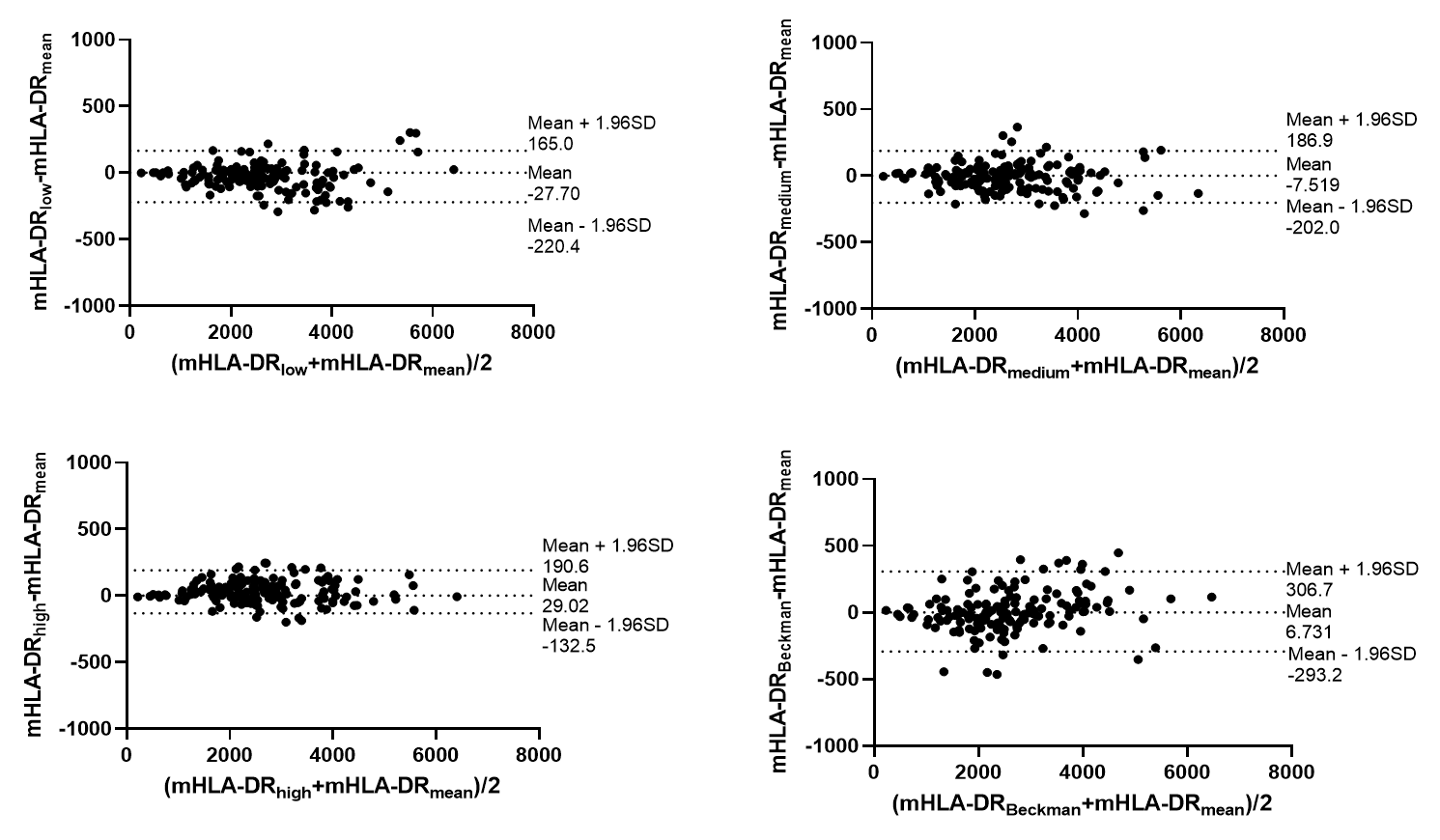


Supplementary Figure 4 The Bland-Altman plot for the four MESFs of mHLA-DR under BD FACSCanto II with high/medium/high voltages and Beckman Coulter DxFlex with proper voltage after removing the outliers. The agreement between the mean value of the four results and each result of mHLA-DR was assessed over the full range. The fixed range was defined as mean ± 1.96SD. mHLA-DR, monocyte HLA-DR; SD, standard deviation; MESF, molecules of equivalent soluble fluorochrome.
